# Integration of 18-FDG PET/CT in the Initial Work-Up to Stage Head and Neck Cancer: Prognostic Significance and Impact on Therapeutic Decision Making

**DOI:** 10.3389/fmed.2020.00273

**Published:** 2020-06-26

**Authors:** Jean-Christophe Leclere, Olivier Delcroix, Jean Rousset, Gerald Valette, Philippe Robin, Catherine Guezennec, Romain Le Pennec, Dorothy M. Gujral, Maelig Abgral, Luc Ollivier, Remi Marianowski, Pierre-Yves Salaun, Ulrike Schick, Ronan Abgral

**Affiliations:** ^1^Department of Head and Neck Surgery, Brest University Hospital, Brest, France; ^2^Department of Nuclear Medicine, Brest University Hospital, Brest, France; ^3^Department of Radiology, Military Hospital Brest, Brest, France; ^4^EA 3878 GETBO IFR 148, University of Western Brittany, Brest, France; ^5^Clinical Oncology Department, Imperial College Healthcare NHS Trust, Charing Cross Hospital, London, United Kingdom; ^6^Department of Cancer and Surgery, Imperial College London, London, United Kingdom; ^7^Department of Radiotherapy, Brest University Hospital, Brest, France

**Keywords:** head and neck cancer, 18-FDG PET/CT, initial assessment, prognostic, therapeutic

## Abstract

**Background:** The objective of this study was to assess the therapeutic and prognostic impact of integrating18F-fluorodeoxyglucose (18-FDG) positron emission tomography (PET)/computed tomography (CT) into work-up (WU) at initial staging of patients with head and neck squamous cell carcinoma (HNSCC).

**Method:** 477 consecutive patients (414M/63F, mean age 62.3 ± 9.7 years) with newly diagnosed HNSCC who underwent pre-treatment 18-FDG PET/CT were retrospectively included. The 18-FDG PET/CT stage (sPET) was compared to the conventional work-up stage (sCWU). A group of cancer specialists determined whether integrating PET/CT into WU at initial staging had an impact on the therapeutic decision, classifying the clinical impact as high (change in therapeutic modality), medium (change in the radiotherapy or surgical procedure), or low (modification of TNM staging and/or detection of synchronous cancer without high or medium impact). Three-year overall survival (OS) was considered as primary endpoint of the prognostic analysis.

**Results:** 18-FDG PET/CT had a clinical impact in 221 patients (46.3%) with a medium or high impact on management in 94 (19.5%) patients. Medium and high impact of 18-FDG PET/CT was statistically equivalent between sCWU-stage I/II and III/IV subgroups (*p* = 0.02). 42 patients were PET/CT-upstaged from early stage I/II to advanced stage III/IV and had a significantly lower 3-year OS than those with concordant CWU and 18-FDG PET/CT early stage (54.8 vs. 82.6%, *p* = 0.001).

**Conclusion:** This study demonstrated that implementing 18-FDG PET/CT in the initial WU of HNSCC provides valuable staging information with a better prognostic stratification. Patient management was modified for any disease stage, even for early stage I-II, with consequences on survival.

## Introduction

Head and neck cancer is the sixth most common malignancy worldwide, with around 800,000 new cases and 320,000 deaths annually (1). These malignancies encompass cancers of the oral cavity, oropharynx, hypopharynx, and larynx and are squamous cell carcinomas (HNSCC) in 90% of the cases. The 5-year survival rate does not exceed 80% for patients with localized disease whereas it decreases to 50% in case of regional lymph node involvement, and to 20% when distant metastasis are present at diagnosis (2).

Surgery or radiation therapy with or without chemotherapy are the cornerstones of treatment, but multimodal management is often required in case of locally advanced disease (3). Hence, accurate cancer staging remains essential to select the appropriate treatment strategy. The gold standard based on conventional work-up (CWU) includes physical examination, endoscopy, computed tomography (CT), and/or magnetic resonance imaging (MRI) of the head and neck to assess the local and regional disease extension. Thoracic CT is also recommended as the lung is the most common site of distant metastasis and synchronous primary cancer (SPC) (3).

The use of 18F-fluorodeoxyglucose (18-FDG) positron emission tomography (PET)/computed tomography (CT) has significantly increased recently in the field of oncology. It allows a whole-body assessment in a single scan with a relatively low radiation exposure (4). Some studies have shown that 18-FDG PET/CT is superior to CWU to assess remote extension and to detect occult SPC (5–8). Nevertheless, pre-therapeutic 18F-FDG PET/CT is currently only recommended to assess distant extension and/or to detect SPC in locally advanced HNSCC and not in early stage cancers (3, 9–11). Modification of staging after integrating 18F-FDG PET/CT as part of the initial WU and the impact on prognosis and clinical management remains also poorly understood and not clearly reported in the literature.

We hypothesized that 18-FDG PET/CT may improve HNSCC staging after initial WU regardless of disease stage, resulting in potential change in patient management.

Therefore, the objective of this study was to assess the impact of the additional information provided by 18F-FDG PET/CT on HNSCC initial staging and whether integrating PETs into WU modify clinical management and prognosis, regardless of the CWU-based staging.

## Materials and Methods

### Population

Patients who underwent a pre-therapeutic 18-FDG PET/CT in our nuclear medicine department for newly diagnosed histologically proven HNSCC between 2004 and 2014 were retrospectively included. All patients (≥ 18 years old) had a complete CWU (clinical examination, panendoscopy, head and neck CT, and/or MRI depending on primary tumor location and thoracic CT) within 4 weeks after diagnosis. Exclusion criteria were previous history of head and neck cancer, treatment started before 18-FDG PET/CT completion, non-squamous cell carcinoma histology, and cervical lymph node metastases of unknown primary tumor. The study was approved by the Institutional Ethics Committee (29BRC18.0012) (RENOVATE ClinicalTrials.gov NCT03841175) and all patients provided written informed consent. The study followed the French General Data Protection Regulation (GDPR).

### PET/CT Imaging

The examinations were performed on several successive PET/CT hybrid machines: Gemini GXLi (Philips© Healthcare, Netherlands) and Biograph-mCT (Siemens©, Erlangen, Germany). Patients were required to be fasting for at least 6 h, and the average blood glucose measured before injection of the tracer was 5.79 ± 0.12 mmol/l. After an intravenous injection of 3–5 MBq/kg of 18-FDG (IBA Molecular Imaging©, Saclay, France), patients remained calm and at rest (bedbound for about 1 h). CT was initially performed in the cranio-caudal direction with a whole-body protocol and injection of iodized contrast material (1.5 mL/kg) (after confirmation of no contraindication to contrast material). Whole-body PET/CT data were acquired in 3D mode and included both emission images (2 to 3 min per step), and transmission images required for attenuation correction. The transmission images were obtained from the X-ray scan data. The emission images were corrected for background noise, random events and reconstructed with and without attenuation correction using the iterative LOR (line of response) RAMLA (row-action maximum likelihood algorithm) method for the Gemini system and the iterative ordered subset expectation maximization (OSEM with point spread function (PSF) modeling and Time-of-Flight (TOF) acquisition capabilities) method for the Biograph system. The PET images were smoothed with a Gaussian filter (full-width at half maximum (FWHM) = 2 mm). The 6-slice Gemini and 40-slice Biograph scanner had, respectively 600 and 700 mm transverse fields of view.

### Staging Assessment

Tumor staging criteria [UICC: Union of International Cancer Control, 7th edition (12)] and the TNM classification [American Joint Committee on Cancer classification (AJCC) 7th edition (13)] in effect during the inclusion period were applied. CWU staging (sCWU) was deduced from clinical and imaging examinations and reviewed at the multidisciplinary team meeting. Following this, staging assessed by 18-FDG PET/CT (sPET) was determined by a single experienced nuclear medicine (OD) blinded to the CWU findings and with only knowledge of the primary tumor location. Visual analysis was used to determine abnormal uptake of 18-FDG, in comparison with the blood pool activity (internal jugular vein) for lymph nodes and the physiological uptake of organs for metastasis (the average background of the organ involved was used for reference). Wider staff review for consensus was undertaken in doubtful situations using a Standard Uptake Value-based semi-quantitative approach by calculating a target-to-background ratio (TBR) by dividing the maximum SUV of the pathological uptake by the mean SUV of the local background activity.

### Clinical Impact

A panel of 3 ENT (Ear, Nose, Throat) cancer specialists [an ENT surgeon (JCL), a radiation oncologist (LO) and a nuclear medicine physician (RA)] convened to retrospectively analyze patient records. The National Comprehensive Cancer Network guidelines in effect at the time of cancer diagnosis were used (3). The panel was asked to define a hypothetical therapeutic strategy, called C-treatment, according to sCWU (whilst blinded to the sPET findings or to the multidisciplinary team meeting decision). The panel was then asked to define a hypothetical therapeutic strategy, called P-treatment, based on sPET (whilst still blinded to the multidisciplinary team meeting decision but not blinded to the C-treatment decision). Subsequently, the different treatments suggested were compared. If the P-treatment was identical to that decided by the multidisciplinary team meeting but differed from the C-treatment, we concluded that sPet altered the treatment strategy. In all other cases, and when the patient had not received the proposed multidisciplinary team treatment, we concluded that sPET did not change treatment.

By analogy with the literature (14–17), impact of sPET on the management plan was classified as: high impact (change in therapeutic modality, e.g., from surgery to radiotherapy and/or in therapeutic objective (curative to palliative), and/or diagnosis of a SPC amenable to curative-intent therapy); medium impact (e.g., change in the radiotherapy target volume or in the surgical procedure); low impact (all modification was limited to staging and/or SPC discovery).

### Survival Analysis

Patients were followed-up for a minimum of 3 years by clinical examination as recommended by guidelines (18). Overall survival (OS) and progression-free survival (PFS) were chosen as primary and secondary endpoints, respectively. OS was defined as the duration of time from diagnosis to death from any cause. PFS was defined as the duration from diagnosis to disease progression, recurrence or death. Patients that were lost to follow-up were censored during the survival analysis.

Downstaging cases involving a medium or high therapeutic impact were noted Des+ (Des = de-escalation), those without medium or high impact noted Des-; upstaging cases involving a medium or high therapeutic impact were noted Esc+ (Esc = escalation), those without a medium or high therapeutic impact noted Esc-.

### Statistical Analysis

The Kaplan-Meier method was used to estimate OS and PFS. The log-rank test was used to compare survival rates according to stage and management impact (19). The Chi-squared 2 test was used to compare the incidence of therapeutic impacts by location and stage, and the TOST equivalence test to compare therapeutic impacts between groups. The level of significance of the *p*-value was 0.05. Statistical analyses were performed using SPSS v25 software (IBM Corp©, Armonk, NY).

## Results

### Population

Four hundred and seventy-seven patients diagnosed between March 2004 and April 2014 were retrospectively included. Of these, 317 (66%) benefited from 18-FDG PET/CT with intra-venous contrast-enhanced infusion. The main characteristics of the study population are summarized in Table 1.

**Table 1:** Patients characteristics (*n* = 477).

**Characteristics**		***n* (%)**
Gender		
	Male	414 (86.8)
	Female	63 (13.2)
Age (average in years ± SD)	62.3 ± 9.7	
Primary location		
	Oral Cavity	99 (20.8)
	Oropharynx	187 (39.2)
	Larynx	103 (21.6)
	Hypopharynx	88 (18.4)
CWU staging		
	Early stages I/II	130 (27.3)
	Advanced stages III/IV	347 (73.7)

According to the oncology network compiling all newly diagnosed HNSCC in the territory, 76% of patients underwent sPET between 2010 and 2014 (available data) for initial staging regardless of stage, and 67% of stage I and II underwent sPET.

Treatment-related features are described according to primary tumor location in Table 2. Of the 430 patients (90%) receiving curative treatment, 257 (60%) received radiotherapy, with or without chemotherapy.

**Table 2:** Treatment-related features according to the primary tumor location.

	**Oral Cavity** ***n* = 99**	**Oropharynx** ***n* = 187**	**Larynx** ***n* = 103**	**Hypopharynx** ***n* = 88**	**Total** ***n* = 477**
Curative treatment	90 (90.9)	172 (92.0)	96 (93.2)	72 (81.8)	430 (90.1)
Surgery	53 (58.9)	54 (31.4)	48 (50.0)	18 (25.0)	173 (40.2)
Radiotherapy	37 (41.1)	118 (68.6)	48 (50.0)	54 (75.0)	257 (59.8)
Palliative treatment	9 (9.1)	15 (8.0)	7 (6.8)	16 (18.2)	47 (9.9)

*Results are in number of patients, percentages in brackets*.

### Follow-Up

Median follow-up ± SD was 31 months (range 0–156). Fourteen (2.9%) of the 477 patients were lost to follow-up in the first year and 12 (2.5%) in the following 2 years. One hundred seventy-nine patients (38%) progressed/relapsed after a median follow-up of 13 months (range 1–62) and 216 (45%) died with a median follow-up of 22 months (range 1–150).

**Figure 1:**
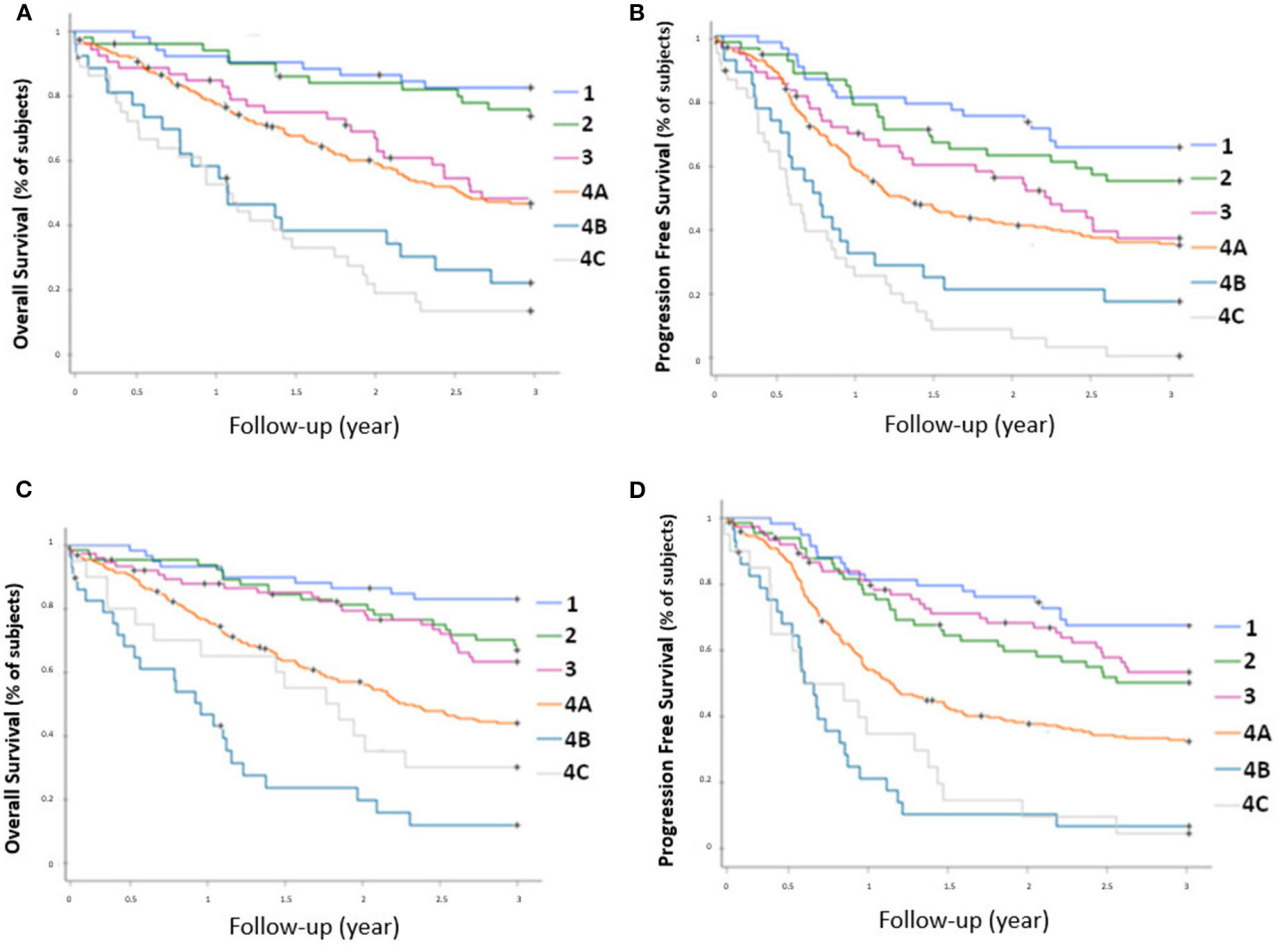
OS rate **(A)** and PFS rate **(B)** for each AJCC stage determined by 18-FDG PET/CT, and OS rate **(C)** and PFS rate **(D)** determined by conventional imaging.

Prognostic stratification according to AJCC classification of sPET and sCWU are presented in Figure 1. A statistically significant association was observed between sPET and both 3-year OS and PFS (*p* < 0.0001). Likewise, a statistically significant association was also observed between sCWU and both 3-year OS and PFS, but with a lower discrimination (crossover or inversion of curves of the stages II vs. III and IVB vs. IVC).

### Clinical Impact

**Figure 2:**
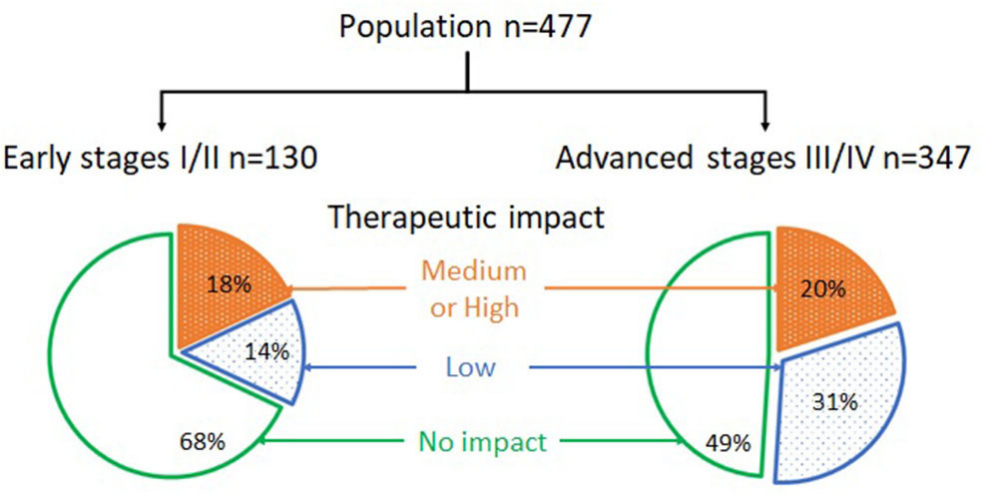
Clinical impact in early and advanced stage tumors.

Data regarding the clinical impact are presented in the Figure 2. Two hundred and twenty-one patients (46.3%) were restaged by sPET and/or detected with an occult CWU SPC, including 56/477 (11.7%) cases of downstaging and 165/477 (34.6%) cases of upstaging.

These changes were mainly related to lymph node status (38.2%) and more rarely to the detection of a SPC (7.3%) or occult metastases (4.5%).

Amongst the 62/477 (13%) patients with one or more SPC (histologically proven and/or confirmed by follow-up), sPET detected 60 cases of which 35 (56%) were undetected by sCWU. SPC were located in the lung, head and neck, esophagus, colon, prostate, liver, breast and stomach in 35.5, 19.4, 17.7, 11.3, 8.1, 3.2, 1.6, and 1.6% of cases, respectively. In addition, there was one case of lymphoma (1.6%). sPET failed to detect only 2 sCWU-detected SPCs−1 hepatocellular carcinoma and 1 lung cancer.

Overall, sPET led to clinical impact in 42 (32.0%) of the 130 sCWU stage I/II patients. This was significantly lower than in the sCWU advanced III/IV stages patients (177/347 = 51.0%, *p* = 0.02). The modification of staging in laryngeal cancers was significantly lower compared to other primary tumor locations, both for upstaging toward an advanced stage (*p* = 0.025) and for overall modification of staging (*p* = 0.014).

#### Medium and High Impact

sPET led to management changes (medium and high impact) in 93/477 patients (19.5%). Forty-four patients (9.2%) had a medium impact, mainly in patients managed by radiotherapy (77%). In 49/477 patients (10.3%), sPET resulted in a high impact. These changes were mainly due to the diagnosis of a SPC (63%) or to the switch from curative to palliative treatment (33%). Medium and high impact changes were significantly more frequent for oral cavity, oropharyngeal, and hypopharyngeal locations than for the larynx (*p* = 0.03).

Amongst the sCWU stage I/II patients, sPET upstaging resulted in a medium or high therapeutic impact in 24 patients (18.4%). This was similar to the sCWU advanced stage III/IV patients (70/347 = 20.1%, *p* = 0.02).

#### Low Impact

One hundred and twenty-eight patients (26.8%) were restaged by sPET and/or detected with an occult CWU SPC, without any medium or high impact.

The results of clinical impact by primary tumor location are detailed in Tables S1, S2 in the Appendix.

### Impact on Survival

#### All Stages

**Figure 3:**
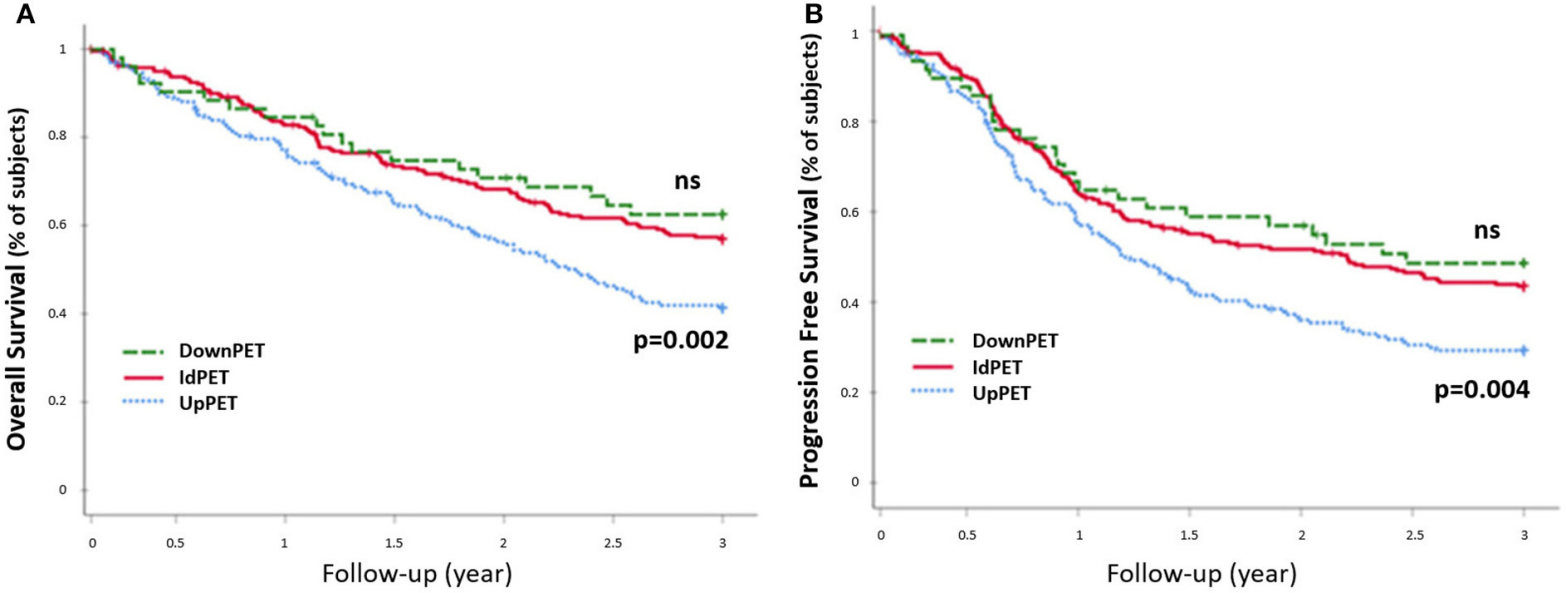
3-year OS rate **(A)** and PFS rete **(B)**, all stages combined (*n* = 477). Patients in upstaging (UpPET) and downstaging (DownPET) were compared with patients with identical 18-FDG PET/CT and CWU stages (IdPET).

**Figure 4:**
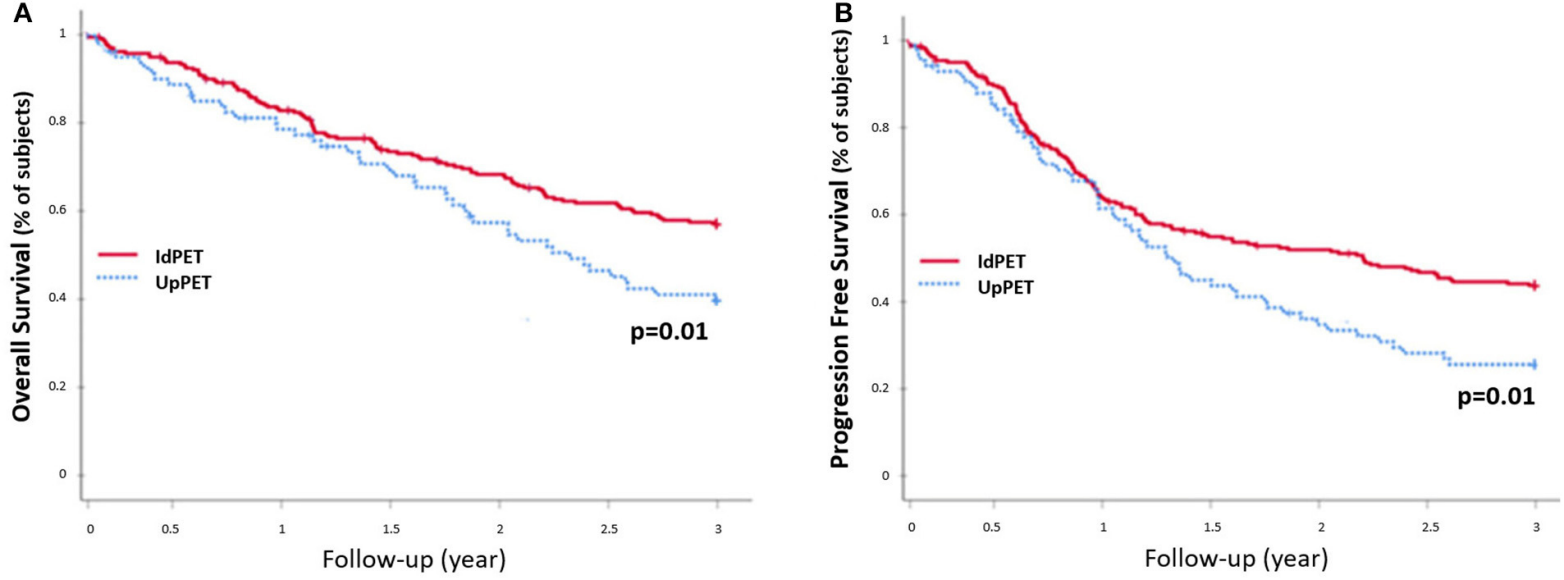
3-year OS rate **(A)** and PFS rate **(B)**, excluding patients with a medium or high therapeutic impact and downstaging (*n* = 330): patients in upstaging (UpPET) have a decreased survival compared to patients with identical 18-FDG PET/CT and CWU stages (IdPET).

Compared to concordant sPET and sCWU patients, sPET upstaged patients had significantly worse 3-year OS survival (44.2 vs. 59.8%; *p* = 0.002) (Figure 3). Excluding patients with medium or high therapeutic impact (in order to avoid the potential consequences of a therapeutic escalation), sPET upstaged patients had significantly lower 3-year OS (38.1 vs. 57.3%; *p* = 0.01) (Figure 4).

#### Early Stages (I/II)

**Figure 5:**
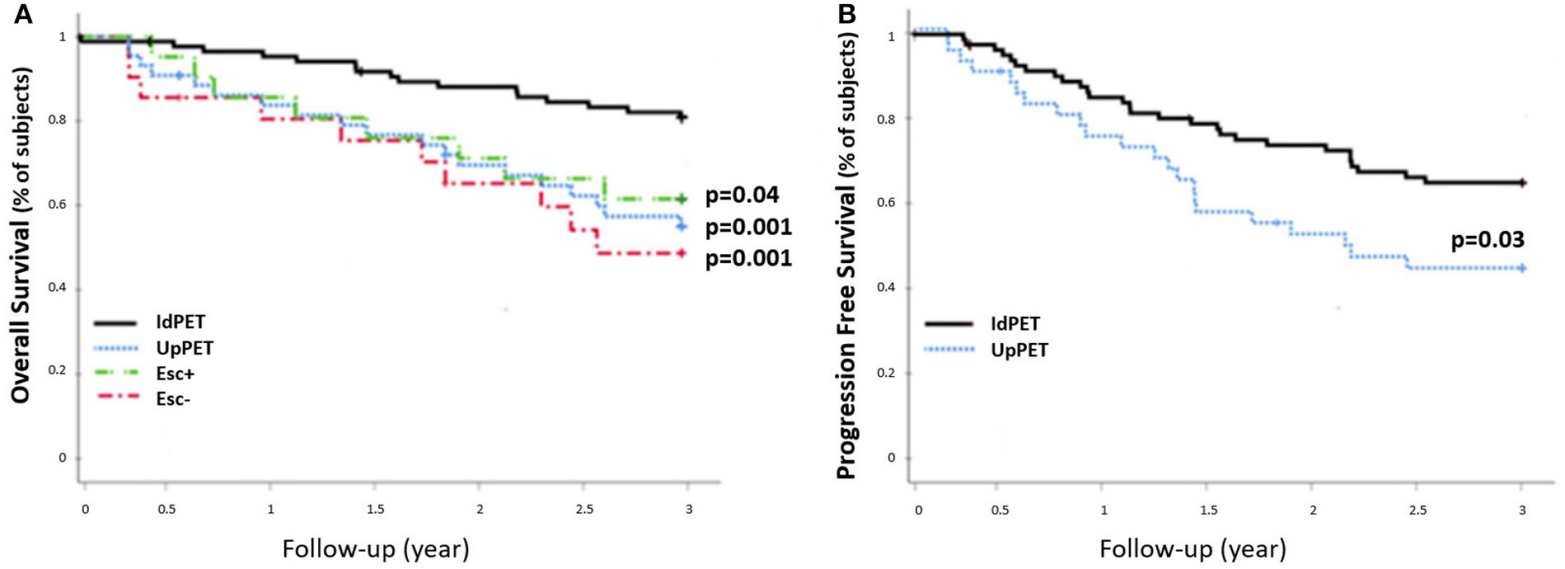
3-year OS rate **(A)** and PFS rate **(B)** of sCWU stage I/II patients without (IdPET, *n* = 88) and with (UpPET, *n* = 42) 18-FDG PET/CT upstaging. Patients for whom the 18-FDG PET/CT led to a therapeutic escalation are noted Esc+ (*n* = 21), the others are noted Esc− (*n* = 21).

The 42 patients sPET upstaged from stage I/II (sCWU) to stage III/IV had significantly lower 3-year OS than those with concordant staging (54.8 vs. 82.6%, *p* = 0.001). This remained significant after excluding those with medium and high therapeutic impact to limit the possible effect of a therapeutic escalation (*n* = 21 Esc-, *p* < 0.01; *n* = 21 Esc+, *p* = 0.04) (Figure 5).

#### Advanced Stages (III/IV)

**Figure 6:**
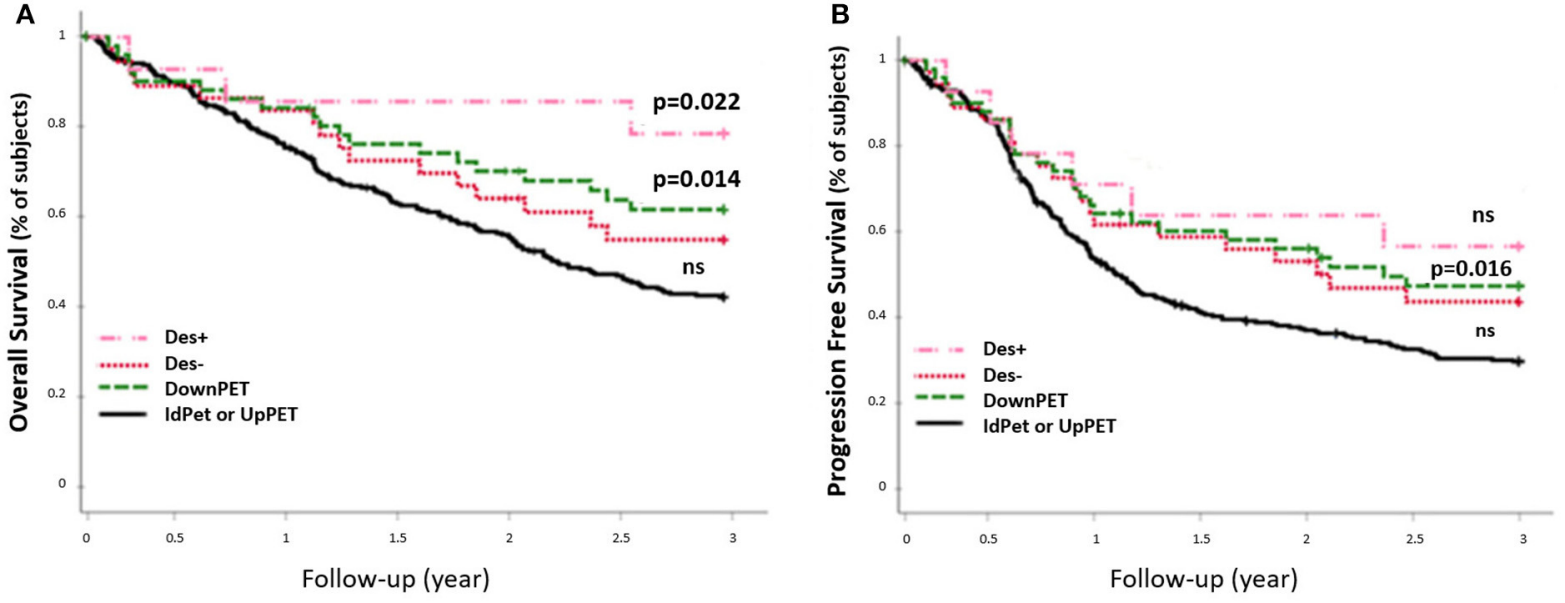
3-year OS rate **(A)** and PFS rate **(B)** of sCWU advanced stage III/IV, with 18-FDG PET/CT downstaging (DownPET, *n* = 54) and concordant staging or upstaging (IdPET or UpPET *n* = 293). Patients for whom the 18-FDG PET/CT led to a therapeutic de-escalation are noted Des+ (*n* = 14), the others are noted Des− (*n* = 40).

The 54 patients who were downstaged with sPET from stage III/IV (sCWU) to stage I/II had significantly higher 3-year OS than those with a concordant sCWU and a sPET advanced stage (64.8 vs. 44.4%, *p* = 0.014) (Figure 6). The results tended to remain significant after excluding patients with medium and high therapeutic impact to limit the possible effect of a therapeutic desescalation (*n* = 40 Des-, *p* = 0.1), whereas OS was significantly higher when therapy was de-escalated (*n* = 14 Des+, *p* = 0.022).

## Discussion

Our study demonstrated the real added value of 18-FDG PET/CT for (i) HNSCC initial staging in addition to the initial WU regardless of clinical stage and (ii) assessing remote disease extension and detection of SPCs with consequential alteration of the CWU-determined management plan. To the best of our knowledge, this study is the largest to assess the management impact of 18-FDG PET/CT in newly diagnosed HNSCC patients.

Foremost, our results suggested superior performance of sPET to assess nodal and metastatic status than sCWU [statistically significant prognostic stratification of sPET (*p* < 0.001)]. In fact, unlike sCWU, sPET strictly respected the prognostic stratification of the AJCC stages (13). sCWU IVB patients had inferior 3-year OS than sCWU IVC patients (11.7 vs. 30%). Conversely, sPET IVB patients had better survival than sPET IVC patients (26.7 vs. 18.5%), closer to the 3-year OS observed in the literature of ~30% (13, 20).

In our series, we found a high rate (46.3%) of modification of staging and/or detection of occult-sCWU SPCs by integrating sPET into initial WU. In comparison, Cacicedo et al. (17) reported a modification of staging rate of 38%, but without including occult-sCWU SPCs detected by sPET. These results are concordant with our modification of staging rate of 40.7%. Moreover, this rate was statistically higher for sCWU advanced stages patients compared to early stages (51 vs. 32% *p* = 0.02). This could be explained by the lower sensitivity of sPET in cN0 patients (without suspicious node), as already demonstrated (20, 21).

The SPC rate in our series was 13%, with 7.3% of occult-sCWU SPCs detected by sPET. These rates are in accordance with the literature: Jones et al. (21) found a SPC rate of 9.1% in a study of 3,436 patients. Moreover, Haerle et al. (22) and Strobel et al. (23) reported detection rates of 6.1 and 7.9%, respectively, in smaller cohorts of patients.

Of the 130 sCWU early stage I/II patients, 29 (22%) were sPET-upstaged to stage III/IV. Furthermore, in this early stage subgroup, 14 occult-sCWU SPCs (10.8%) were detected by sPET, altering management and prognosis (24). sPET led to a therapeutic escalation (medium or high treatment modification) in 24 patients (18.5%). This rate was statistically equivalent for advanced stage (stage III/IV) patients (*p* = 0.02), highlighting the sPET diagnostic accuracy even for early stages.

In the same way, amongst sCWU advanced stage III/IV patients, sPET-downstaged patients with therapeutic de-escalation (medium or high impact) with significantly better 3-year OS (78.6 vs. 44.4%; *p* = 0.02), whereas sPET-downstaged patients without therapeutic de-escalation had a slightly lower 3-year OS but also tended to be significantly higher than non-downstaged patients (*p* = 0.1). It can be assumed that the impact of therapeutic de-escalation is positive on survival. However, it could not be excluded that this lack of significance in downstaged patients without de-escalation could be attributed to decreased number of patients and/or selection bias. Our other hypothesis is that treatment-related morbidity and mortality is higher for downstaged patients without therapeutic de-escalation.

In our study, therapeutic impact assessment was carried out using strict methodology, and only the certainties of treatment modification were retained. Probable changes in treatment but without certainty were not retained, especially when P-treatment was different from C-treatment and different from the treatment proposed by the multi-disciplinary team of experts (*n* = 6). Our medium or high treatment modification rate was 19.5% (*n* = 477). In the literature, this rate is variable: assessed at 13.7% (*n* = 233) for Lonneux et al., 15.7% (*n* = 248) for Ryu et al., 26.1% (*n* = 84) for Cacicedo et al., 33.8% (*n* = 71) for Scott et al., and up to 40% (*n* = 35) for Connell et al. (7, 14–17). This variability is probably due to different elements. First, the use of external radiotherapy varied widely from 25 to 74%, depending on the series. In our study, 59.8% of patients were curatively treated with external radiotherapy, and 77% of medium management changes were related to this treatment. Indeed, the large majority of sPET modification of staging in our series concerned regional lymph nodes involvement. This could have easily impacted radiotherapy treatment planning (25, 26). Second, patient management modification rates were lower in the more recent studies, probably related to improvement in imaging techniques. The more frequent use of MRI has probably helped to improve the sensitivity of the CWU. Third, the recent technical innovation of 18-FDG PET/CT may also improve lesion detection and the general use of contrast-enhanced combined CT may decrease the number of false positives results with resultant improved physician interpretation. Finally, the recent advent of dynamic whole-body PET/CT imaging supported by the latest clinical PET systems may also allow the use of more quantitative markers, beyond SUV and static uptake metrics, to further improve the clinical usefulness of sPET with respect to sCWU (27, 28).

Given our large patient cohort, we were able to perform subgroup analysis based on the localization of the primary tumor, unlike other series in the literature. Thus, our results demonstrated sPET outperformed sCWU for initial evaluation of oropharyngeal, hypopharyngeal, and oral cavity cancers with ~20% change in treatment plan (medium or high). This rate was significantly lower for laryngeal cancers (13.6%, *p* = 0.03), which was probably due to the low nodal involvement rate of this primary tumor location.

Our study had several limitations. First, the retrospective design requires validation by a randomized prospective trial. Second, Human Papilloma Virus (HPV)/p16 status was not routinely determined for HNSCC in our institution during the inclusion period. Therefore, this could lead to heterogeneity within our cohort with different prognoses of HPV+ and HPV- patients according to the 8th TNM classification, which was published after our last patient inclusion (29). Third, during the 10-year inclusion period, 18-FDG PET/CT examinations were performed on 3 different systems of different generations. However, this corresponds to a real-life study, the results of which could be applicable in different centers. Fourth, only two-thirds of PET scans were contrast-enhanced and, at worst, it decreases the diagnostic performance of 18-FDG PET/CT vs. CWU. Finally, this study was not designed to assess the cost-effectiveness of 18-FDG PET/CT in initial routine imaging workup.

Current guidelines optionally recommend the use of 18-FDG PET/CT for early CWU stages I/II to look for synchronous carcinoma that may modify the treatment plan (11). Our findings suggest that the systematic implementation of 18-FDG PET/CT in the initial WU of HNSCC, regardless of disease stage, could improve the staging and the treatment plan with significant consequences on survival outcome.

In conclusion, our study demonstrated that implementation of 18-FDG PET/CT in the initial WU of HNSCC provides valuable staging information with better prognostic stratification. Patient management was improved for any disease stage, even early stages I-II. Amongst patients with advanced disease, about half were restaged, and about a third of patients with early disease stage were restaged. In total, the management plan was altered for about 20% of patients. Furthermore, 18-FDG PET/CT modification of staging and treatment changes were significantly associated with OS. In addition, our study confirmed the interest of 18-FDG PET/CT for SPC detection, regardless of the stage.

## Data Availability Statement

The datasets generated for this study are available on request to the corresponding author.

## Ethics Statement

The study was approved by the Ethics Committee Brest University Hospital (29BRC18.0012) (RENOVATE ClinicalTrials.gov NCT03841175) and all patients signed a written consent form. The study follows the French General Data Protection Regulation (GDPR).

## Author Contributions

RA, J-CL, and OD are the guarantors of the paper and realized statistics. RA, JR, GV, J-CL, and OD designed the study. RM, J-CL, and GV ensured inclusion and follow-up of patients. RA, OD, and JR managed imaging procedures. DG revised English language. RA, LO, and J-CL analyzed patient records. RA, MA, OD, and J-CL analyzed the data. All authors contributed to the article and approved the submitted version.

## Conflict of Interest

The authors declare that the research was conducted in the absence of any commercial or financial relationships that could be construed as a potential conflict of interest.
