# Supplementary material for: Integration of 18-FDG PET/CT in the Initial Work-Up to Stage Head and Neck Cancer: Prognostic Significance and Impact on Therapeutic Decision Making
Source: Front Med (Lausanne). 2020 Jun 26;7:273. doi: 10.3389/fmed.2020.00273 (PMC7344296; doi:10.3389/fmed.2020.00273)
Supplement: Supplementary file 1 [file Table_1.docx]

|  |  | Minor therapeutic impact | | | | | | | | | | | |
| --- | --- | --- | --- | --- | --- | --- | --- | --- | --- | --- | --- | --- | --- |
|  |  | on N | | on M | | SPC | | Total | | upstaging | | downstaging | |
| Oral cavity | n=99 | 38 | (38,4) | 4 | (4,0) | 9 | (9,1) | 43 | (43,4) | 33 | (76,7) | 10 | (23,3) |
| Oropharynx | n=187 | 77 | (41,2) | 10 | (5,3) | 9 | (4,8) | 92 | (49,2) | 67 | (72,8) | 25 | (27,2) |
| Larynx | n=103 | 34 | (33,0) | 3 | (2,9) | 9 | (8,7) | 44 | (42,7) | 30 | (68,2) | 14 | (31,8) |
| Hypopharynx | n=88 | 33 | (37,5) | 5 | (5,7) | 8 | (9,1) | 42 | (47,7) | 35 | (83,3) | 7 | (16,7) |
| Total | n=477 | 182 | (38,2) | 22 | (4,6) | 35 | (7,3) | 221 | (46,3) | 165 | (74,7) | 56 | (25,3) |

Table S1 Type of minor therapeutic impact, by modifying lymph node status (N), metastatic status (M), or synchronous cancer discovery. The types of impact are differentiated into upstaging and downstaging. The percentages in brackets are given in relation to the total number of patients.
