# Supplementary material for: Integration of 18-FDG PET/CT in the Initial Work-Up to Stage Head and Neck Cancer: Prognostic Significance and Impact on Therapeutic Decision Making
Source: Front Med (Lausanne). 2020 Jun 26;7:273. doi: 10.3389/fmed.2020.00273 (PMC7344296; doi:10.3389/fmed.2020.00273)
Supplement: Supplementary file 2 [file Table_2.docx]

|  | | |  | Therapeutic impact | | | | | | | | | | | | |
| --- | --- | --- | --- | --- | --- | --- | --- | --- | --- | --- | --- | --- | --- | --- | --- | --- |
|  |  | Moderate | | | | | | | Major | | | | | Moderate + Major | | |
|  |  | RT+ | | RT- | CR+ | CR- | Total Moderate | | >Pallia. | >Cur. | Traitement modif. | Total Major | |  |  |  |
| Orale Cavity | n=99 | 4 | | 1 | 2 | 1 | 8 | (8,1) | 0 | 1 | 9 | 10 | (10,1) | 18 | (18,2) | |
| Oropharynx | n=187 | 15 | | 4 | 4 | 1 | 24 | (12,8) | 9 | 1 | 10 | 20 | (10,7) | 44 | (23,5) | |
| Larynx | n=103 | 2 | | 2 | 0 | 0 | 4 | (3,9) | 3 | 0 | 7 | 10 | (9,7) | 14 | (13,6) | |
| Hypopharynx | n=88 | 4 | | 2 | 2 | 0 | 8 | (9,1) | 4 | 0 | 5 | 9 | (10,2) | 17 | (19,3) | |
| Total | n=477 | 25 | | 9 | 8 | 2 | 44 | (9,2) | 16 | 2 | 31 | 49 | (10,3) | 93 | (19,5) | |

Table S2 Distribution of moderate and major therapeutic impacts by primary tumour location. (RT+ : increase in GTV and/or dose, RT- : decrease in GTV and/or dose, CR+ : increase in surgical resection, CR- decrease in surgical resection, >pallia. change from curative to palliative treatment, >cur. change from palliative to curative treatment, treatment modification: change from radiotherapy to surgery or vice versa, or SPC.
